# Effects of Acupuncture on CCL2 and CXCL8 Expression and the Subset of uNK Cells in Rats with Embryo Implantation Failure

**DOI:** 10.1155/2013/678390

**Published:** 2013-12-15

**Authors:** Weina Gao, Xiao Tang, Zhenyan Chen, Yue Guo, Lijun Wang, Mingmin Zhang, Guangying Huang

**Affiliations:** Institute of Integrated Traditional Chinese and Western Medicine, Tongji Hospital, Tongji Medical College, Huazhong University of Science and Technology, Wuhan, Hubei 430030, China

## Abstract

The present study was designed to investigate the efficacy and mechanism of acupuncture treatment on embryo implantation failure in rats. The pregnant rats were randomized into normal group (N), implantation failure group (M), acupuncture treatment group (A), and progestin treatment group (W). The embryo implantation failure model was established by mifepristone. Efficacy of acupuncture treatment was evaluated by the number of implanted embryos. The expression of CCL2 and CXCL8 and the subset of uterine natural killer cells in the endometrium were detected. We demonstrated that the number of implanted embryos was dramatically reduced after mifepristone (M group) treatment, while the acupuncture (A group) and progestin (W group) treatments significantly rescued impaired embryo implantation. The protein and mRNA expressions of CCL2 and CXCL8 were significantly reduced by mifepristone treatment, but the attenuated expression of CCL2 and CXCL8 was markedly reversed by acupuncture or progestin treatment. More importantly, acupuncture and progestin could markedly increase the subset of uNK cells in rats with embryo implantation failure. These evidences suggest that acupuncture is able to modulate the endometrial immune microenvironment and thus improve embryo implantation in pregnant rats, which provides solid experimental evidence for the curative effect of acupuncture treatment on infertility.

## 1. Introduction

Embryo development to the blastocyst stage, successful implantation into the uterine endometrium, and the formation of a functional placenta are essential steps during pregnancy. Successful embryo implantation and healthy placental development allow the fetus to acquire maternal nutrient supply and maintain its growth and development until parturition. As a mucosal tissue, the endometrium must protect against infection and create a unique immune microenviroment to allow the implantation of a semiallogeneic embryo and decidual artery remodeling for placental development [1].

Chemokines are a large superfamily of structurally and functionally related cytokines with chemotactic activity targeted at specific leukocyte populations. Chemokines play important roles in both homing of leukocytes to specific regions within a tissue and as potent activators of leukocytes. It is clear that chemokines are important determinants of successful implantation and placentation by their function as leukocyte chemoattractants, influence on trophoblast migration, and by additional functions such as modulation of cell proliferation and modification of adhesion molecule expression [2]. CXCL8, an CXC chemokine, is produced by chorion trophoblast [3] and decidua [4], and is localized to the perivascular cells of the placental villi during the first trimester and at term [5]. It is not only a potent chemoattractant and activator of neutrophils and T-lymphocytes, but also is known to have several other functions, including serving as a mediator for vascular smooth muscle migration [6] and an angiogenic factor [7]. Monocyte chemoattractant protein belongs to the CC-chemokines subfamily and is a potent chemoattractant and activator of monocytes, macrophages, T-cells, basophils, mast cells, and natural killer cells. CCL2 is released by a number of cell types such as endothelial cells, fibroblasts, and endometrial cells. Denison et al. demonstrated that significantly higher levels of CCL2 are released from peripheral blood monocytes in pregnant women compared to nonpregnant women [8]. CCL2 is present at high concentrations in the extraembryonic coelomic fluid during the first trimester of pregnancy [9]. The expression of CCL2 and its receptor CCR2 can be upregulated by pregnancy-associated hormones, for example, estrogen, progesterone, and human choriogonadotropin (HCG), which suggests that the interaction of CCR2 and CCL2 may be involved in regulation of decidual stromal cell functions [10].

During pregnancy, a large number of leukocytes accumulate in the endometrium. These cells are a source of growth factors, cytokines, and proteases and create local microenvironments permissive to tissue remodeling. These leukocytes have been implicated in fetal-maternal interactions at the implantation site [11]. Uterine natural killer (uNK) cells are the most abundant terminally differentiated lymphocyte population found in mesometrial decidua during early pregnancy in women and rodents [12, 13]. In humans, uNK cells (CD3−, CD56+, and CD16−) constitute up to 70% of all decidual leukocytes during early gestation. These cells begin to infiltrate the endometrium on day LH +3, specifically accumulate around spiral arterioles and areas of decidualized stroma, and are present in the decidua until the second trimester of pregnancy [12]. Their presence is consistent with the period of trophoblast invasion. In mice and rats, uNK cells have been referred to in the past as “granulated metrial gland cells” (GMG) or “large granular lymphocytes.” uNK cells congregate at the implantation sites between the placenta and the metrial gland or mesometrial lymphoid aggregate of pregnancy (MLAp) and form a unique structure located in the mesometrial triangle during rodent pregnancy [14]. NKR-P1, as a signal transduction molecule, is expressed on all rat large granular lymphocytes with NK activity [15]. Recent evidence from mice lacking NK cells suggested that these specialized immune cells play important roles in the invasion of foetal trophoblast cells and spiral arteriole remodeling [16].

To date, assisted reproductive techniques (ART), such as IVF-embryo transfer (IVF-ET) and intracytoplasmic sperm injection (ICSI), have effectively solved some preimplantation problems, including the obstruction of the oviduct and fertilization failure; however, the clinical success rate of pregnancy is still unsatisfactory and the cost of IVF-ET is high, which not only causes high emotional and financial distress for many patients but also upsets clinicians. So, how to improve successful rate of IVF-ET has become a critical problem for clinicians. Acupuncture is widely used for the treatment of acute and chronic disorders in China. Recently, several studies have shown that acupuncture can improve the rate of pregnancy in patients treated with ART. A previous study from our team demonstrated that acupuncture can increase the implantation rate up to four times compared to rats with embryo implantation failure [17]. Anderson et al. also suggested that acupuncture improves the success rate of IVF and may be a safe adjuvant therapy for IVF patients [18]. As mentioned above, CCL2, CXCL8, and uNK cells play important roles in embryo implantation and placentation during pregnancy, so whether acupuncture can modulate the expression of these chemokines and the subset of uNK cells has been rarely studied.

Acupuncture, as an important part of traditional Chinese medicine therapy, is based on the theory that there are channels (meridians) within the body where “qi” and “blood” circulate. The whole body is connected by meridians. Along these meridians, there are specific points (acupoints). By needling the acupoints, acupuncture can regulate the flow of “qi” and “blood” in the meridian system, correct dysfunction of organs in the body, and restore their normal functions. According the theory of Traditional Chinese medicine (TCM), “Housanli” acupoint is one of the most important acupoints in the body and can effectively prevent diseases and prolong life. “Sanyinjiao” acupoint is widely used to treat the diseases of obstetrics and gynecology. So, these two acupoints in rat were selected for treating embryo implantation failure.

Progesterone induces decidualization of endometrium and reduces the activity of uterus, whereas mifepristone inhibits the function of progesterone by binding to the progesterone receptor. In the current study, we used mifepristone to generate a rat embryo implantation dysfunction model and progestin as a positive model for acupuncture effect. We then investigated whether acupuncture affects the expression of CCL2 and CXCL8 and the subset of uNK cells. This study will provide valuable experimental evidence regarding the curative efficacy of acupuncture on infertility.

## 2. Materials and Methods

### 2.1. Animals

Female Wistar rats (10–12 weeks old, weight 205 ± 18 g) and adult male Wistar rats (weight 250–300 g) were obtained from the Center for Disease Control and Prevention of Hubei province. We followed The Guidelines for the Care and Use of Animals in Research enforced by Hubei Municipal Science and Technology Commission. All protocols were approved by the Institutional Animal Care and Ethics Committee of Tongji Medical College, Huazhong University of Science and Technology. The rats were caged in a standard barrier system with a 12-hour light : 12-hour dark cycle.

### 2.2. Reagents

The mifepristone tablets (Beijing Zizhu Pharmaceutical Co., Ltd., Beijing, China) and progestin (Zhejiang Xianju Pharmaceutical Co., Ltd., Hangzhou, China) were provided by Tongji Hospital. Collagenase type IV (C5138), hyaluronidase (H3506) and phenylmethanesulfonyl fluoride solution (93482) were purchased from Sigma (St. Louis, MO, USA). EZ-Sep Mouse (DKW33-R0100) was purchased from Dakewe Biotech Co., Ltd. (Beijing, China). RPMI 1640 medium (SH30809.01B) was from Thermo Fisher Scientific Co., Ltd. (Beijing, China). FBS was purchased from Hangzhou Sijiqing Biological Engineering Materials Co., Ltd. (Hangzhou, China). HRP-goat anti-rabbit IgG (H + L) conjugate (ZB-2301) and DAB coloring reagent kit used for immunohistochemistry were products of Beijing Zhongshan Biotech Co., Ltd. (Beijing, China). RIPA lysis buffer (P0013B), BCA protein assay kits (P0010S), and BeyoECL Plus (P0018) were from Beyotime Institute of Biotech (Haimen, China). Cocktail protease inhibitor was from Wuhan Gugeshengwu Technology Co., Ltd. (Wuhan, China). HRP-goat anti-rabbit IgG (sc-2030) and goat or rabbit anti-rat actin polyclonal antibody (sc-1616R) were from Santa Cruz Biotechnology, Inc. (Santa Cruz, CA, USA). The total RNA extract reagent (Trizol), PrimeScript RT Reagent Kit Perfect Real Time (TaKaRa Code: DRR037A), and SYBR Premix Ex Taq (TaKaRa Code: DRR041) were from TaKaRa Biotechnology (Dalian) Co., Ltd. (Dalian, China). Rabbit anti-rat CCL2 (ab7202), rabbit anti-rat CXCL8 (ab7747) were purchased from Abcam (Cambridge, MA, USA). FITC anti-rat CD3 Antibody (201403), Alexa Fluor 647 anti-rat CD161 antibody (203110), Alexa Fluor 647 mouse IgG1, and *κ* Isotype Ctrl antibody (400135) were from BioLegend Ltd. (San Diego, CA, USA). Nucleic acid/protein analyzer (DU730, Beckman Coulter, Inc., Fullerton, CA, USA); Mastercycler gradient PCR apparatus (Eppendorf Company, Hamburg, Germany); Nikon Micro-imaging System (TE2000-U, Tokyo, Japan); Applied Biosystems StepOne Real-Time PCR System (Applied Biosystems, Invitrogen, Grand Island, NY, USA); Microplate reader (BioTek Synergy2, Winooski, VT, USA); FACSCalibur (BD Bioscience, San Jose, CA, USA).

### 2.3. Experimental Design

After 1-week acclimation, male and female rats were mated at 6 PM, and Day 1 of pregnancy was defined by vaginal smears on the second morning. The pregnant rats were randomly divided into one of four groups: normal group (N), mifepristone group (implantation failure group) (M), acupuncture treatment group (A), and progestin treatment group (W). The rats in each group were equally randomized into a D6 group (*n* = 12), a D8 group (*n* = 12), and a D10 group (*n* = 12) according to the time of sample collection. The embryo implantation failure model and treatment were performed as described previously [17]. Briefly, the rats in groups M, A, and W were treated with mifepristone solution at 5.5 mg/kg by neck subcutaneous injection on D1 at 9 AM, while the rats in group N were injected with the same amount of sesame oil. “Housanli” (ST36) and “Sanyinjiao” (SP6) were selected for acupuncture. A self-made restraint was used to restrict the activity of the rats. The rats in group A were secured with the self-made restraint and then received acupuncture at 3PM every day from D1 to the time of death. Acupuncture treatment was performed for 25 min every day. Correspondingly, the rats in group W were given progestin at 40 mg/kg by intramuscular injection.

### 2.4. Samples Collection

The rats were killed by cervical dislocation under 1% pentobarbital sodium on days 6, 8, and 10 after mating. The uteri were collected and the number of fetuses was counted. The uteri from each group were equally divided into two subgroups (*n* = 6). The intact uterus of one subgroup was immediately placed into 1640 medium (supplemented with 10% fetus bovine serum (FBS) for the analysis of uNK cells. Partial uterus tissue from the other subgroup with implantation sites was fixed in 4% paraformaldehyde solution for paraffin imbedding, while the remaining tissues were stored at −80°C for RNA extraction and protein detection.

### 2.5. The Isolation of Endometrial Lymphocytes and Flow Cytometric Analysis of uNK Cells

Fresh uterus was washed completely in PBS supplemented with 50 *μ*g/mL gentamicin. The uterus cavity was cut open along the antimesometrial side. Under a dissection microscope, all fetuses were discarded, and the endometrium with implantation sites and the intact placenta was reserved. Collected tissues were finely minced into ∼1 mm^3^ pieces. These pieces were enzymatically digested in RPMI 1640 medium containing 0.1% collagenase type IV and 0.1% hyaluronidase in an agitated water bath at 37°C for 2 hours. The mixture of tissue was passed through a stainless steel mesh into medium using the syringe plunger and centrifuged at 1500 g for 5 min at room temperature. The cell pellet was resuspended in RPMI 1640 medium with 10% FCS, carefully layered on lymphocyte separation medium, and then centrifuged at 2000 g for 30 min at 22°C. The white lymphocyte cell layer was aspirated from the interface and harvested. A trypan blue exclusion test identified that more than 95% of the isolated cells were viable, and then the cells were resuspended to a density of 10^4^ cells/mL. Cell surface staining with FITC-labeled anti-rat CD3 monoclonal antibody and Alexa Fluor 647-labeled anti-rat CD161 monoclonal antibodies was accomplished using a 30 min incubation at RT in the dark. As a control, cells were stained with the corresponding isotype-matched antibody. The cells were resuspended in 100 *μ*L PBS buffer and then analyzed with FACSCalibur. Instrument compensation was set in each experiment using single-color stained samples. Data were analyzed by using WinMDI2.9 software.

### 2.6. Immunohistochemistry

Paraffin sections (5 *μ*m) were kept in an oven at 60°C for 1 hr. These sections were deparaffinized and rehydrated through degraded ethanol. Antigen retrieval was performed by incubating the sections in 0.01 M citrate buffer (pH 6.0) at 98°C for 20 min. Endogenous hydrogen peroxidase activity was quenched using 3% H_2_O_2_. Nonspecific binding was prevented by preincubation of tissue sections with 5% bovine serum albumin (BSA). Primary antibodies were diluted in PBS (CCL2, 1 : 150; CXCL8, 1 : 40) and incubated with tissue sections overnight at 4°C. For the negative control, slides were incubated in PBS. The sections were incubated with HRP labeled goat anti-rabbit IgG then incubated with fresh 3,3′-diaminobenzidine and counterstained with Harris haematoxylin. The tissue staining was observed under a Nikon Micro-imaging System and average staining intensity of each picture was measured with Image-Pro Plus 6.0 software.

### 2.7. Western Blot Analysis

Endometrial implantation sites without a fetus were homogenized in RIPA lysis buffer then centrifuged at 12,000 g for 20 min at 4°C to remove insoluble material. The supernatant was collected and stored at −80°C. Protein concentration was quantified with the BCA protein assay kit. Protein lysates were heated at 98°C for 10 min, resolved by 12% (*β*-actin) or 15% (CCL2 and CXCL8) SDS-PAGE and electrotransferred to nitrocellulose membranes using a semidry transfer apparatus. The membranes were then blocked with 5% nonfat milk in PBST for 2 hrs at room temperature then incubated with diluted antibodies (*β*-actin 1 : 200; CCL2 1 : 500; CXCL8 1 : 100) overnight at 4°C. The membranes were incubated with horseradish peroxidase-conjugated anti-rabbit secondary antibody (1 : 20,000; room temperature for 1 hr). Peroxidase activity was visualized with BeyoECL Plus according to the manufacturer's instructions. Signal was visualized using the Epson Imaging System. The quantitation of band density was performed using the Quantity One software. Results were calculated as a ratio (protein of interest/*β*-actin).

### 2.8. Real-Time Fluorescent Quantitative Polymerase Chain Reaction (PCR)

Endometrial implantation sites without a fetus were dissociated with Trizol reagent, according to the manufacturer's instructions. RNA purity and concentration were measured by a nucleic acid/protein analyzer. Complementary DNA (cDNA) was synthesized in master-cycler gradient PCR apparatus using PrimeScript RT Reagent Kit following the standard protocol. The primers were designed according to published sequences (Table 1). The cDNA was stored at −20°C for batched analyses. PCR was performed in triplicate in a 20 *μ*L reaction volume including 10 *μ*L of SYBR Premix Ex Taq (2x), 2 *μ*L of DNA sample, 10 *μ*M of each primer, 0.4 *μ*L of ROX Reference Dye (50x), and 6.8 *μ*L of dH_2_O. Then, amplification was carried out on an Applied Biosystems StepOne Real-Time PCR System. *β*-Actin was used as an internal standard to control the variability in amplification. The data were analyzed by the 2^−ΔΔct^ method.

### 2.9. Statistical Analysis

All the experimental data were expressed as mean ± standard deviation (SD). One-way analysis of variance (ANOVA) followed by LSD test was used for data with equal variances assumed. When data with equal variances were not assumed, statistical significance was determined using ANOVA followed by Dunnett's T3 test. Statistical significance was set at the level of *P* < 0.05.

## 3. Results

### 3.1. Embryo Implantation in the Rat Uterus

**Figure 1:**
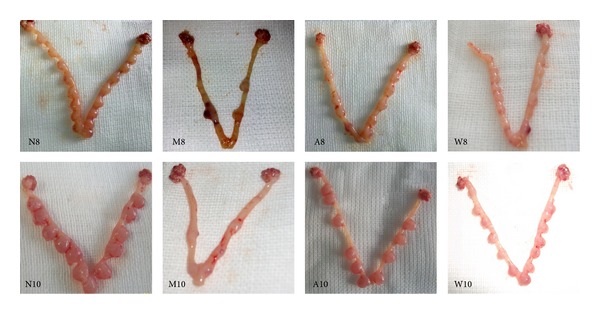
Morphology and number of implanted embryo in rat uterus on D8 and D10 of pregnancy. N: normal group; M: embryo implantation failure group; A: acupuncture treatment group; W: progestin treatment group. These abbreviations were applied to all figures and tables.

The uteri were examined for the number of implanted embryos as well as their morphological status. Representative images and statistical analysis are shown in Figure 1 and Table 2, respectively. Compared with other three groups, the size of embryos was smaller and distribution of implantation sites was more disorderly and asymmetric on D8 and D10 in the mifepristone group. The number of implanted embryos in the mifepristone group was remarkably less than that in normal group (*P* < 0.001). However, compared with the mifepristone group, the number of embryos was obviously higher in the acupuncture treatment group (*P* < 0.01) and progestin treatment group (*P* < 0.001).

### 3.2. Immunohistochemical Analysis of  CCL2 and CXCL8 in Rat Endometrium

**Figure 2:**
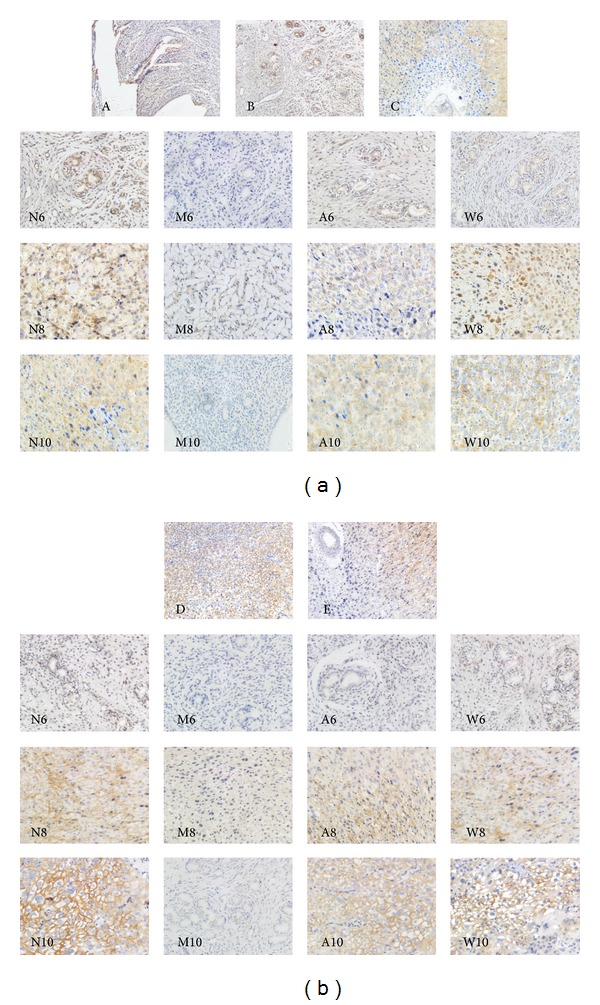
Immunohistochemical localization of CCL2 (a) and CXCL8 (b) in rat endometrium on D6, D8, and D10 of pregnancy. The positive staining was in brown. (a) CCL2 staining in rat endometrium: panel A showed the expression of CCL2 in the endometrial luminal epithelium; panel B represented the staining of CCL2 in the endometrial glandular epithelium and vascular endothelium; panel C was the expression of CCL2 in decidualized stroma; N6-W10 displayed the expression of CCL2 in rat decidualized region on D6, D8, and D10 of pregnancy. (b) CXCL8 localization in rat endometrium: N6-W6 was weak CXCL8 expression in rat endometrial glandular epithelium on D6 of pregnancy; N8-W8 presented CXCL8 localization in rat decidualized region on D8 of pregnancy; CXCL8 was expressed in placental district on N10, A10, and W10, but in endometrial glandular epithelium on M10. Except for A, B, C, D, and E (×100), magnification for other graphs was ×200.

**Figure 3:**
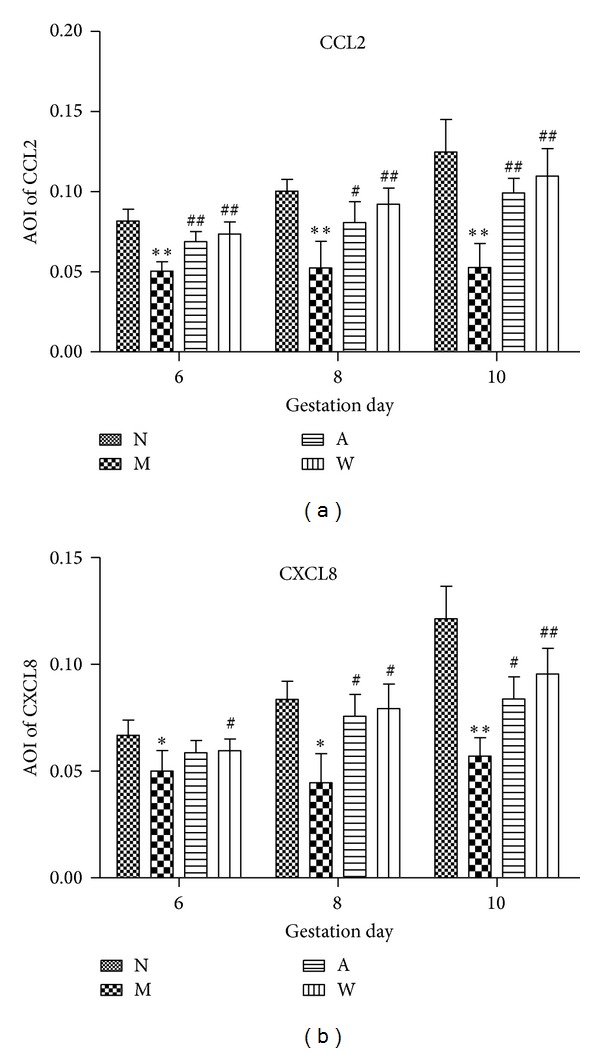
Quantitation of CCL2 (a) and CXCL8 (b) immunostaining in rat endometrium on D6, D8, and D10 of pregnancy. AOI is average optical intensity. Data are presented as mean ± SD (*n* = 6). *P < 0.05, **P < 0.01 compared with N; ^#^ *P* < 0.05, ^##^ *P* < 0.01 compared with M.

CCL2 protein was primarily localized in the luminal epithelium, the glandular epithelium, the decidual stroma, and the vascular endothelium. The endometrial CCL2 protein level in the mifepristone group was found to be significantly lower than that in the normal group on D6, D8, and D10 (*P* < 0.01). Importantly, compared with the mifepristone group, there was a remarkable increase in CCL2 protein level after acupuncture treatment on D6, D10 and progestin treatment on D6, D8, and D10 (*P* < 0.01). A small increase was seen in rat endometrium CCL2 after acupuncture treatment on D8 (*P* < 0.05) (Figures 2(a) and 3(a)). CXCL8 protein was slightly expressed in the luminal epithelium and the glandular epithelium on D6; however, the expression of CXCL8 was abundant in the placental region on D8 and D10. Compared with the mifepristone group, the endometrial CXCL8 protein level was dramatically stimulated in the normal group and progestin treatment group on D10 (*P* < 0.01). Similarly, a marked increase of CXCL8 protein was observed in normal group and progestin treatment group on D6 and D8, as well as acupuncture treatment group on D8 and D10 (*P* < 0.05) (Figures 2(b) and 3(b)).

### 3.3. Western Blot Analysis of CCL2 and CXCL8 in Rat Endometrium

**Figure 4:**
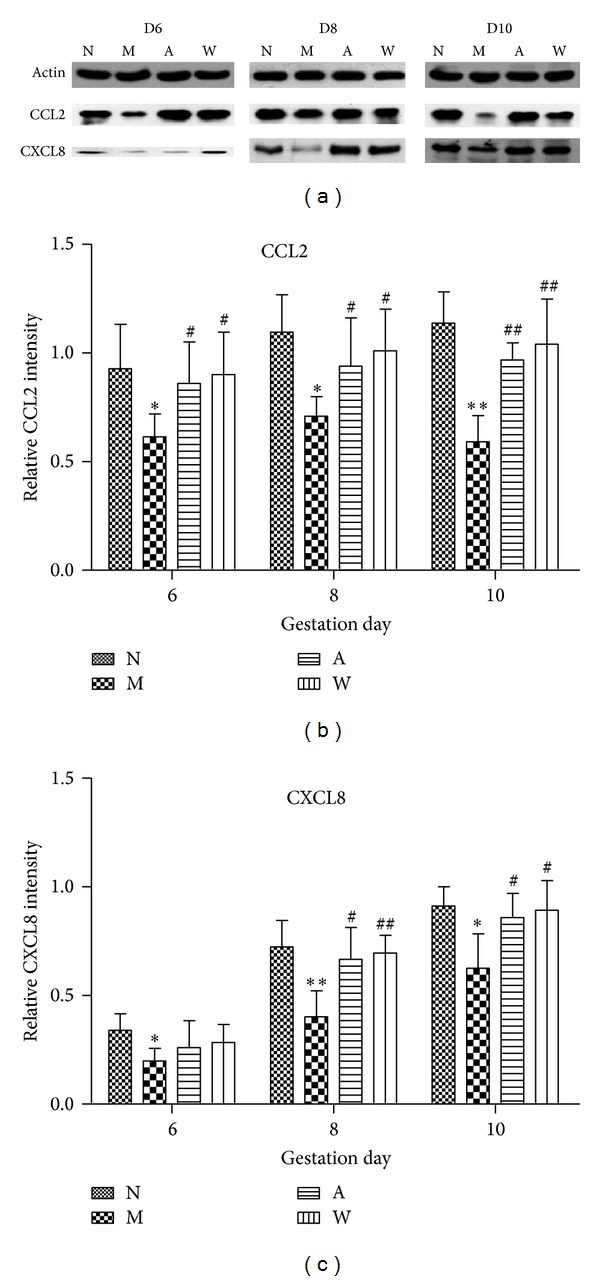
The expression of CCL2 and CXCL8 proteins in rat endometrium on D6, D8, and D10 of pregnancy. (a) Representative blots of CCL2 and CXCL8. (b and c) Statistical analysis of CCL2 (b) and CXCL8 (c) in rat endometrium. The relative intensity was determined by the ratio of interested protein to its corresponding internal control (*β*-actin) as measured by densitometry. Data are presented as mean ± SD (*n* = 6). *P < 0.05, **P < 0.01 compared with N; ^#^ *P* < 0.05, ^##^ *P* < 0.01 compared with M.

The quantitative change in endometrial CCL2 and CXCL8 expression was evaluated by Western blot, as shown in Figure 4. Compared with the normal group, the expression of CCL2 protein was significantly reduced in the mifepristone group on D6, D8 (*P* < 0.05), and D10 (*P* < 0.01). Compared to the mifepristone group, a significant increase in CCL2 protein expression was observed in the acupuncture treatment and progestin treatment groups on D6, D8 (*P* < 0.05), and D10 (*P* < 0.01) (Figures 4(a) and 4(b)). CXCL8 protein level in the endometrium was increased in a time-dependent manner; the higher expression was observed on D8 and D10. Compared to the normal group, the expression of CXCL8 protein was notably reduced in the mifepristone group on D6 (*P* < 0.05), D8 (*P* < 0.01), and D10 (*P* < 0.05). However, the CXCL8 protein level was dramatically elevated in the acupuncture treatment group on D8 and D10 (*P* < 0.05) and progestin treatment group on D8 (*P* < 0.01), and D10 (*P* < 0.05) as compared to that in the mifepristone group (Figures 4(a) and 4(c)). No significant difference in CXCL8 expression was found among acupuncture, progestin, and mifepristone treatments on D6 (Figure 4(c)).

### 3.4. mRNA Expression of CCL2 and CXCL8 in Rat Endometrium

**Figure 5:**
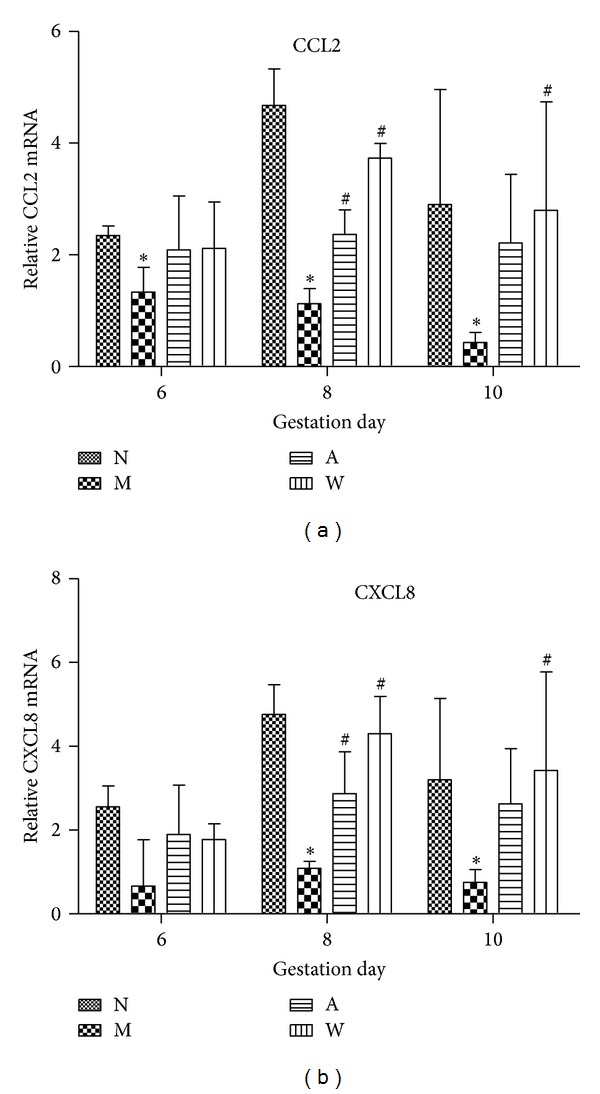
The expression of CCL2 (a) and CXCL8 (b) mRNA in rat endometrium on D6, D8, and D10 of pregnancy. Relative quantitation of CCL2 and CXCL8 expression was determined by the ratio to *β*-actin expression. Results were represented as means ± SD (*n* = 6). *P < 0.05, **P < 0.01 compared with N; ^#^ *P* < 0.05, ^##^ *P* < 0.01 compared with M.

The relative abundance of CCL2 and CXCL8 was examined by real-time PCR data (Figure 5). Compared to the normal group, CCL2 mRNA expression was markedly decreased in the mifepristone group on D6, D8, and D10 (*P* < 0.05, Figure 5(a)). Compared to the mifepristone group, the expression of CCL2 mRNA was elevated in the acupuncture treatment and progestin treatment groups on D6, D8, and D10, but only several comparisons (acupuncture treatment group, *P* < 0.05 compared to mifepristone group on D8; progestin treatment group, *P* < 0.05 compared to mifepristone group on D8 and D10) reached statistical significance (Figure 5(a)). There was no significant difference in the expression of CXCL8 mRNA among the four groups on D6. However, a lower level of CXCL8 mRNA was identified in the mifepristone group compared to the normal group on D8 and D10 (*P* < 0.05, Figure 5(b)). More importantly, acupuncture and progestin treatments could rescue the attenuated CXCL8 mRNA following mifepristone treatment on D8. Progestin treatment also sharply increased CXCL8 mRNA after mifepristone treatment on D10 (*P* < 0.05, Figure 5(b)).

### 3.5. Percentage of uNK (CD3−CD161+) Cells in Rat Endometrium

**Figure 6:**
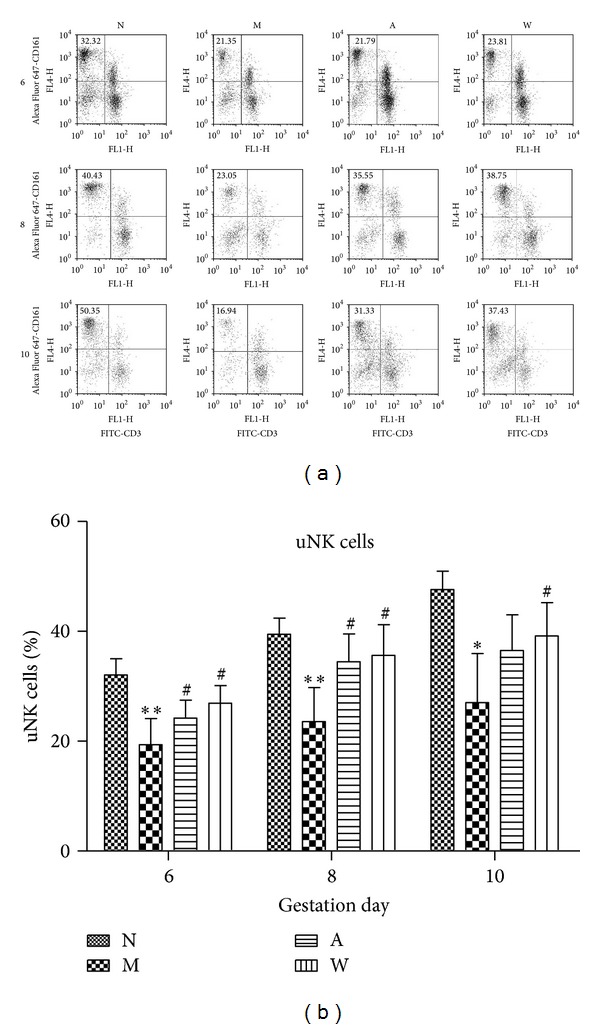
Flow cytometric analysis of uNK cell subset in rat endometrium on D6, D8, and D10 of pregnancy. (a) The representative graphs of 2-color flow cytometric analysis of uNK cell subset (gated to lymphocytes). (b) Statistical analysis of uNK (CD3−CD161+) cell subset in rat endometrium. The percentage of uNK cell subset was presented as mean ± SD (*n* = 6). *P < 0.05, **P < 0.01 compared to N; ^#^ *P* < 0.05, ^##^ *P* < 0.01 compared to group M.

Compared to the normal group, a lower percentage of CD3−CD161+ cells was observed after mifepristone treatment (*P* < 0.001) on D6 and D8. The subset of CD3−CD161+ cells was notably elevated after acupuncture and progestin treatment as compared to that after mifepristone treatment alone on D6 and D8 (*P* < 0.05, Figures 6(a) and 6(b)). Moreover, the subset of CD3−CD161+ cells was dramatically reduced after mifepristone treatment on D10 (*P* < 0.05), but there was no significant change in the proportion of CD3−CD161+ cells between mifepristone and acupuncture treatments on D10 (Figures 6(a) and 6(b)).

## 4. Discussion

Successful embryo implantation and perfect placentation are indispensable for establishing a pregnancy. The accomplishment of these two processes requires a functionally normal embryo at the blastocyst stage and a receptive endometrium, as well as adequate communication between them under hormonal stimulation. This dialogue is regulated accurately by various chemokines and immune cells, which are required to maintain immune-privileged sites at the fetomaternal interface. Recently, acupuncture has been extensively used to treat infertility, including ovulatory dysfunction, in vitro fertilization and embryo transfer (IVF-ET), and male infertility. We hypothesize that acupuncture may modulate the immune microenviroment at the maternal-fetal interface, increasing the chance for a successful implantation.

In this study, we observed that the number of embryos was remarkably decreased after mifepristone treatment on D6, D8, and D10, and the embryo was smaller and embryonic distribution of implantation sites was asymmetric. However, the acupuncture treatment could significantly increase the number of implanted embryo. These results indicated that the model of embryonic implantation failure was successfully established by mifepristone treatment and acupuncture therapy can contribute to embryo implantation during gestation. More importantly, we confirmed the involvement of chemokines and immune cells through the examinations of CXCL8 and CCL2 expression and the subsets of uNK cells in rat implantation sites. We also demonstrated that acupuncture indeed increased the expression of CXCL8 and CCL2 and the subset of uNK in rats with embryonic implantation failure.

In rodents, actual implantation of blastocyst into endometrium occurs 6-7 days after fertilization [19], and, if successful, normal vascular remodeling occurs after gd8.5 and is completed by gd10.5, once placental structure is completed and placental blood flow begins [20]. Invasion of trophoblast into the decidua and myometrium, and the remodeling of the spiral arteries are important characteristics of hemochorial placentation. Replacement of the maternal vessel wall by trophoblasts markedly alters the vascular conductance and results in increased blood flow to the intervillous space, which is required for normal fetal growth and development. If the trophoblast invasion cannot proceed, the transformation of maternal vessels will not be complete, which results in serious clinical diseases such as preeclampsia, intrauterine growth retardation [21].

Previous studies suggest that trophoblast invasion is regulated by the interaction of autocrine factors from the trophoblasts and paracrine factors from the uterus. The secretion of matrix metalloproteinases (MMP) from trophoblasts [22] is a key event. Once the extracellular matrices of the host's tissues are digested, trophoblast invasion is completed. Some studies have suggested that CXCL8 is involved in endometrial receptivity and embryonic implantation [23, 24]. CXCL8 stimulates first-trimester extravillous trophoblast cells invading into the decidua by increasing the secretion of MMP-2 [25], and plays unique roles in endometrial angiogenesis, apoptosis, proliferation, and differentiation, which is essential for successful fetal-placental development [26]. Prior studies have shown that downregulation of CXCL8 in the placenta may be responsible for fetal loss and fetal growth retardation [27]. In the present study, immunohistochemical experiment showed that CXCL8 is mainly expressed in trophoblast, decidual cells, glandular, and luminal epithelium. The endometrial CXCL8 mRNA and protein expressions on D6, D8, and D10 were remarkably higher in normal rats but were significantly reduced by mifepristone treatment. Moreover, acupuncture and progestin treatment could rescue CXCL8 expression, which is consistent with the increased number of implanted embryo. These data suggest that CXCL8 is essential for pregnancy. CXCL8 was stably increased from D6 to D10 in all groups except mifepristone groups, which further suggests that CXCL8 is not only necessary for normal embryo implantation but it is also importantly essential for placental development. Acupuncture could promote trophoblast invasion and placentation by upregulating the expression of CXCL8.

The prevailing theory supports the balance between a T-helper 1 (TH1) and TH2 response occuring at the maternal-fetal interface. The initial Th1 response allows the embryo to implant while the shift to a Th2 response manages endocrine and immune communication that ensures successful implantation [28]. Prior data have shown that CCL2 can promote Th2 polarization and sustain the Th2-dominant environment, and that CCL2-deficient mice are present with defective Th2 immunity [29]. In our experiments, endometrial CCL2 was present at a low level after mifepristone treatment, which fits with the observation of fewer implanted embryos. Moreover, acupuncture and progestin treatments could markedly upregulate CCL2 expression compared to mifepristone treatment, which implies that acupuncture treatment can facilitate the function of a TH2 immune response at the maternal-fetal interface during the implantation phase, and thereby promote embryo implantation. Other researchers showed that CCL2 has powerful growth-promoting and angiogenic properties [26, 30]. van Mourik et al. also pointed out that CCL2 can mediate chemotaxis of macrophages in decidua or trophoblast [31]. A large number of macrophages are observed at the implantation site throughout pregnancy, which contributes to endometrial cyclical development, endometrial trophoblast interaction, and endometrial tissue regeneration by secretion of the proinflammatory cytokines, such as TNF-alpha, IL-6, and LIF (leukemia inhibitor factor) [32, 33]. In our study, except in the endometrial decidual cells, glandular and luminal epithelium, CCL2 was also observed in vascular endothelial cells of endometrial arterial wall. These results suggest that acupuncture may contribute to the remolding of endometrial spiral artery or placentation by upregulating expression of CCL2.

In rodents, uNK cells emerge only during pregnancy. About 10% of uNK cells are present in the lumens of decidual vessels, particularly small capillaries. Almost 25% of uNK cells infiltrate into arterial walls and the remainder associates with decidual cells [20, 34]. Guimond et al. demonstrated that uNK cells modify the spiral arterial branches of the uterine arteries using a NK-deficient mouse strain [35]. uNK cells may manipulate the maternal immune response to the fetal allograft, as well as trophoblast invasion and placenta formation, by modulating expression of cytokines [36]. Mouse uNK cells also express angiogenic molecules, such as vascular endothelial growth factor (VEGF), placental growth factor (PIGF), and angiopoietin 2 (ANG2) for angiogenesis and maternal vasculature growth into implantation region [37]. In addition, some evidence has shown that decidual uNK cells influence EVT invasion, potentially by increasing the expression of MMP-9, which is regarded as a key enzyme in the degradation of endometrial basement membrane [38]. In this study, we detected the percentage of CD3−CD161+ NK cells at the rat implantation site by flow cytometry. Our results demonstrated that the proportion of CD3−CD161+ NK cells was clearly reduced in rats with embryo implantation failure, while acupuncture and progestin treatments could markedly increase the subset of CD3−CD161+ NK cells after mifepristone treatment. These data further confirm that uNK cells are vital for embryo implantation and placentation.

In our study, we demonstrated that acupuncture promoted the expression of CCL2 and CXCL8 and increased the subset of uNK cells. Meanwhile, some evidence demonstrated that the upregulation of endometrial CCL2 and CXCL8 expression and uNK cell subset is associated with production of progesterone [7, 23, 39]. Stener-Victorin et al. demonstrated that acupuncture may influence the hypothalamic pituitary-ovarian axis by modulating central endorphin production and secretion, which mediates the release of hypothalamic GnRH and pituitary secretion of gonadotropin [40]. Therefore, we hypothesized that when needles are penetrated into skin and muscle, the peripheral nervous system likely transmits signals to the brain, where the gonadal hormone is regulated by Hypothalamus-Pituitary-Gonadal and -Adrenal axes. Although the progesterone level was not measured in this study, we suppose that the regulatory effects of acupuncture on the expression of CCL2 and CXCL8 and the subset of uNK cells may involve progesterone due to the similar results from progestin treatment.

In conclusion, acupuncture may contribute to embryo implantation and placentation in rat by upregulating the expression of CCL2 and CXCL8 and the subset of uNK cells at maternal-fetal boundary. This study provides new experimental evidence supporting modulation of the nerve-endocrine-immune system by acupuncture and suggests that this therapy may be a novel tool for infertility.

## Figures and Tables

**Table 1:** The primer sequences for real-time PCR.

Gene	Primer	Sequence
*β*-actin	Forward	5′-GGAGATTACTGCCCTGGCTCCTA-3′
	Reverse	5′-GACTCATCGTACTCCTGCTTGCTG-3′
CCL2	Forward	5′-CTATGCAGGTCTCTGTCACGCTTC-3′
	Reverse	5′-CAGCCGACTCATTGGGATCA-3′
CXCL8	Forward	5′-CTCCAGCCACACTCCAACAGA-3′
	Reverse	5′-CACCCTAACACAAAACACGAT-3′

**Table 2:** Implantation sites on D8 and D10 of pregnancy (mean ± SD).

Group	Number of rats	Implantation sites
N	24	12.25 ± 1.67
M	24	4.58 ± 3.30*
A	24	8.17 ± 2.50^#^
W	24	9.25 ± 2.25^##^

The data were expressed as mean ± standard deviation (SD).

*P < 0.001 compared with group N; ^##^ *P* < 0.001,^#^ *P* < 0.01 compared with group M.
